# Intraspecific Functional Trait Response to Advanced Snowmelt Suggests Increase of Growth Potential but Decrease of Seed Production in Snowbed Plant Species

**DOI:** 10.3389/fpls.2019.00289

**Published:** 2019-03-14

**Authors:** Rita Tonin, Renato Gerdol, Marcello Tomaselli, Alessandro Petraglia, Michele Carbognani, Camilla Wellstein

**Affiliations:** ^1^Faculty of Science and Technology, Free University of Bozen-Bolzano, Bolzano, Italy; ^2^Department of Life Sciences and Biotechnology, University of Ferrara, Ferrara, Italy; ^3^Department of Chemistry, Life Sciences and Environmental Sustainability, University of Parma, Parma, Italy

**Keywords:** climate change, plant functional traits, intraspecific trait variability, alpine environment, snowmelt

## Abstract

In ecological theory, it is currently unclear if intraspecific trait responses to environmental variation are shared across plant species. We use one of the strongest environmental variations in alpine ecosystems, i.e., advanced snowmelt due to climate warming, to answer this question for alpine snowbed plants. Snowbeds are extreme habitats where long-lasting snow cover represents the key environmental factor affecting plant life. Intraspecific variation in plant functional traits is a key to understanding the performance and vulnerability of species in a rapidly changing environment. We sampled snowbed species after an above-average warm winter to assess their phenotypic adjustment to advanced snowmelt, based on differences in the natural snowmelt dynamics with magnitudes reflecting predicted future warming. We measured nine functional traits related to plant growth and reproduction in seven vascular species, comparing snowbeds of early and late snowmelt across four snowbed sites in the southern Alps in Italy. The early snowbeds provide a proxy for the advanced snowmelt caused by climatic warming. Seed production was reduced under advanced snowmelt in all seed-forming snowbed species. Higher specific leaf area (SLA) and lower leaf dry matter content (LDMC) were indicative of improved growth potential in most seed-forming species under advanced snowmelt. We conclude, first, that in the short term, advanced snowmelt can improve snowbed species’ growth potential. However, in the long term, results from other studies hint at increasing competition in case of ongoing improvement of conditions for plant growth under continued future climate warming, representing a risk for snowbed species. Second, a lower seed production can negatively affect the seed rain. A reduction of propagule pressure can be crucial in a context of loss of the present snowbed sites and the formation of new ones at higher altitudes along with climate warming. Finally, our findings encourage using plant functional traits at the intraspecific level across species as a tool to understand the future ecological challenges of plants in changing environments.

## Introduction

In the alpine environment, climate change has a dramatic influence on plant life. Rising temperatures are altering the timing of one of the most important determinants of plant distribution, i.e., the growing season and the related phenology (Walter and Breckle, 2002; Chuine, 2010; Matteodo et al., 2016). In this context, strong ecological differences are created by alterations in the timing of the melting of the snow cover because it triggers phenology at the verge of plant life. Responses of plants to environmental modifications are first visible in the adjustment of their functional traits within the reaction norm. However, it is not clear whether the adjustment of trait values is a key for trait-based predictions of species performance in plant communities (Roscher et al., 2018) as individual plant species may differ in their responses to environmental changes (Klanderud, 2005). Recently, the importance of intraspecific functional variability for better understanding species’ ecological fate and community dynamics has been recognized in the ecological literature (Ackerly and Cornwell, 2007; Albert et al., 2011; Violle et al., 2012; Wellstein et al., 2013; Jung et al., 2014; Sides et al., 2014; Escudero and Valladares, 2016; Chalmandrier et al., 2017; Roscher et al., 2018). Intraspecific trait variability can be genetically determined or be the result of the variation of trait phenotype under the direct effect of the environment. The phenotypic modification of a trait may result in different abilities of a plant to cope with new environmental conditions driving rapid plants’ reaction through morphological and physiological adjustment (Nicotra et al., 2010; Wellstein et al., 2013; Garnier et al., 2016). Defining magnitude and direction of phenotypic adjustments within a species will help to formulate hypotheses about the fate of species under climatic and environmental changes (Nicotra et al., 2010; Liancourt et al., 2015).

In this context, the particular vulnerability of alpine snowbeds to climate warming makes this ecosystem particularly suitable for the study of the effect of climate change on phenotypic variation of alpine plant species’ functional traits. Alpine snowbeds are ecologically extreme habitats, situated in areas covered with snow during most of the year. Snow cover is the main environmental factor controlling plant life in snowbeds. Indeed, long-lasting snow cover ensures stable temperatures during winter (Körner, 2003; Björk and Molau, 2007; Pignatti and Pignatti, 2014), protects plants from frost damage (Heegaard, 2002; Shimono and Kudo, 2003; Björk and Molau, 2007), controls soil water content, and determines the length of the growing season in relation to snowmelt date (Körner, 2003; Choler, 2005; Keller et al., 2005; Schöb et al., 2009). Snowbed plant species are highly specialized and well adapted to extreme environmental conditions (Billings and Mooney, 1968; Körner, 2003; Schöb et al., 2008; Hülber et al., 2011; Pignatti and Pignatti, 2014). They usually are very small in size and characterized by high relative growth rate and low carbon investment per unit leaf area to deal with the short growing season (Schöb et al., 2008). Phenology and growth in snowbed species are both closely related to snow persistence and snowmelt timing, that influence soil nutrient availability and soil water content (Venn and Morgan, 2007; Carbognani et al., 2012; Lluent et al., 2013; Livensperger et al., 2016). Snowbed vegetation is particularly sensitive to predicted changes in snow cover under climate warming (Schöb et al., 2008; Baptist et al., 2010; Carbognani et al., 2012, 2014a; Matteodo et al., 2016). Snow cover thickness and duration are strictly linked to air temperature during winter and spring, and to winter precipitation (Heegaard, 2002; Körner, 2003). The majority of future climate models forecasts rising temperatures during the twenty-first century which will imply a significant decrease in snow cover at all elevations in the mountains (Beniston et al., 2003; Watson and Haeberli, 2004; ACIA, 2005; Giorgi and Lionello, 2008; Kotlarski et al., 2012; Steger et al., 2013; Gobiet et al., 2014; Pachauri et al., 2014; Klein et al., 2016). Although some models forecast increasing precipitation in winter, higher fraction of winter precipitation is predicted to fall as rain instead of snow (Giorgi and Lionello, 2008; Gobiet et al., 2014), thus resulting in thinner and consequently less insulating snowpack (Wipf et al., 2009). Higher temperature and reduced snow cover will lead to an earlier snowmelt date that will anticipate the beginning of the growing season of snowbed species (Wipf et al., 2009; Sedlacek et al., 2015). Advanced snowmelt can trigger different mechanisms, i.e., (i) prolonging the duration of the growing season which could enhance plant growth (Theurillat and Guisan, 2001; Winkler et al., 2018), (ii) exposing vegetation to dangerous spring frost events (Inouye, 2008; Wipf et al., 2009; Baptist et al., 2010; Gerdol et al., 2013; Wheeler et al., 2015), (iii) improving fertility of nutrient-poor soils through enhanced mineralization rates (Billings and Bliss, 1959; Stanton et al., 1994; Körner, 2003) and (iv) reducing soil water content (Galen and Stanton, 1991; Giménez-Benavides et al., 2007; Venn et al., 2011; Winkler et al., 2018). Indeed, snowbed soils are subjected to high rates of water loss because of low field capacity coupled with high solar irradiance that enhances evaporation (Isard, 1986; Björk and Molau, 2007). Whereas snowbed vegetation may currently experience dry conditions at the middle or at the end of the growing season only, earlier snowmelt timing may extend drought stress toward the beginning of the growing season (Inouye, 2008; Venn and Morgan, 2009).

The effects of climate change on snowbeds have been extensively investigated in terms of changes in vegetation cover, community composition, species distribution (Galen and Stanton, 1995; Heegaard, 2002; Schöb et al., 2008, 2009; Hülber et al., 2011; Carbognani et al., 2012, 2014b; Sandvik and Odland, 2014; Matteodo et al., 2016) and phenological alterations (Walker et al., 1995; Sandvik and Totland, 2000; Hülber et al., 2010; Dorji et al., 2013; Petraglia et al., 2014a; Carbognani et al., 2016, 2018), sometimes through the analysis of functional diversity and community-weighted trait means (Venn et al., 2011; Pickering et al., 2014; Komac et al., 2015). Few studies have so far focused on the phenotypic variability of the functional traits of snowbed species (Kudo et al., 1999; Baptist et al., 2010; Komac et al., 2015; Sedlacek et al., 2015). In this study, we collected snowbed species after an above-average warm and dry winter (Colucci et al., 2017) to assess the effects of advanced snowmelt on seven representative snowbed plant species through the analysis of phenotypic variation of functional traits. The functional traits considered include both vegetative and reproductive traits that have been found to show plastic adjustment to environmental conditions (Nicotra et al., 2010; Wellstein et al., 2013; Jung et al., 2014; Liancourt et al., 2015; Chelli et al., 2017). These functional traits are related to important plant functions such as photosynthetic capacity, growth; and ultimately, competitive ability, reproduction and survival (Kudo et al., 1999; Baptist et al., 2010; Lluent et al., 2013; Pérez-Harguindeguy et al., 2013; Liancourt et al., 2015). Our study was directed to answer the following questions:

(i) Do snowbed species exhibit an intraspecific response to advanced snowmelt conditions in terms of functional traits?
(ii) Does this intraspecific traits adjustment show a common trend across different snowbed plant species and sites?

## Materials and Methods

### Study Areas and Species

**FIGURE 1:**
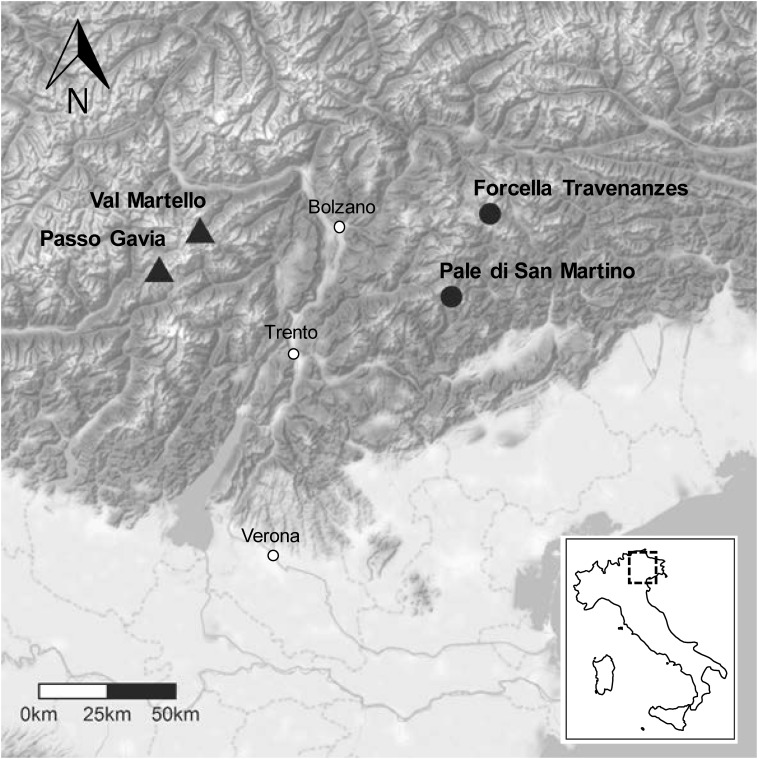
Location of the four snowbed sites in the Italian eastern Alps. Circles and triangles represent calcareous and siliceous sites, respectively.

Four snowbed sites located in the south-eastern Alps of Italy were investigated, two sites on calcareous bedrock and two on siliceous bedrock (Figure 1, see Supplementary Figure S1 for the detailed locations of the sites). The two calcareous sites were selected from snowbeds reported in Pignatti and Pignatti (2014), following the criteria of (i) representativeness of snowbed community and (ii) accessibility of the site. The selected calcareous sites are located at Forcella Travenanzes (46°31^′^ N, 12°1^′^ E, 2450 m, Province of Belluno) and on Pale di San Martino (46°16^′^ N, 11°50^′^ E, 2500 m, Province of Trento). These snowbeds are characterized by vegetation dominated by *Salix retusa* L. and *Salix reticulata* L. (Ellenberg, 1988; Grabherr and Mucina, 1993; Tomaselli et al., 2006; Pignatti and Pignatti, 2014). Less information about siliceous snowbed sites was available. Therefore, we selected siliceous sites with known representation of siliceous snowbed communities. These sites are located close to Passo Gavia (46°20^′^ N, 10°29^′^ E, 2651 m, Province of Sondrio) and in the upper Val Martello (46°28^′^ N, 10°40^′^ E, 2600 m, Province of Bolzano-Bozen). These snowbeds are characterized by vegetation dominated by *Salix herbacea* L. and by the moss *Polytrichastrum sexangulare* (Brid.) G. L. Sm. (Ellenberg, 1988; Grabherr and Mucina, 1993; Carbognani et al., 2014b).

Climatic data were obtained from weather stations close to the sites at almost the same elevation, with the exception of the Solda-Madriccio station located about 200 m above the Val Martello site. At Forcella Travenanzes, the mean annual temperature is -1°C, with an average minimum of -16°C and an average maximum of 13.5°C (data from Ra Valles station, 2592 m). At Pale di San Martino, mean annual temperature is 0.5°C. The average minimum and maximum are -19°C and 15.5°C, respectively (data from Rifugio Rosetta station, 2581 m). At Passo Gavia, mean annual temperature is -1.4°C. The average minimum is -10°C and the average maximum is 9.3°C (data from Passo Gavia, 2651 m, according to WorldClim datasets at a spatial resolution of 30 arc second; Hijmans et al., 2005). At Val Martello, mean annual temperature is -2°C with an average minimum of -23.7°C and a maximum of 15.9°C (data from Solda-Madriccio, 2825 m). Mean total annual precipitation is similar at the four sites, ranging from 1000 mm at Val Martello, to 1054 mm at Pale di San Martino, and 1150 mm at Passo Gavia. No data are available for Forcella Travenanzes. However, mean total annual precipitation at the Passo Falzarego station (2090 m, ca 350 m below the Forcella Travenanzes site) is 1230 mm.

From character and accompanying species of the snowbed communities (Ellenberg, 1988; Grabherr and Mucina, 1993; Pignatti and Pignatti, 2014) which are also the most frequent ones, we selected seven vascular plant species that were present in at least two of both of our calcareous and siliceous snowbed study sites: the two dwarf shrubs *Salix herbacea* and *S. retusa* as character species of the two associations of calcareous and siliceous bedrock, the grass *Poa alpina* L., and the forbs *Veronica alpina* L., *Gnaphalium supinum* L., *Leucanthemopsis alpina* (L.) Heywood and *Polygonum viviparum* L. [ = *Bistorta vivipara* (L.) S. Gray] (see Table 1 and Supplementary Table S1).

**Table 1:** Results of linear mixed effect models for differences between early and late snowmelt areas of single plant traits for each study species across the four study sites.

		Trait								
		
Species	N. of sites	H	LA	SLA	LDMC	LNC	LPC	N:P	SN	SM
*Poa alpina*	4	ns	ns	ns	L^∗^	E^∗∗∗^	ns	E^†^	L^∗∗∗^	E^∗∗^
*Salix herbacea*	3	ns	ns	ns	ns	ns	L^∗∗∗^	E^∗∗∗^	L^∗∗^	ns
*Salix retusa*	2	L^∗∗∗^	L^∗∗∗^	E^∗∗∗^	ns	L^∗∗^	ns	ns	L^†^	ns
*Veronica alpina*	3	E^†^	ns	E^∗∗^	L^∗∗∗^	ns	ns	E^∗^	L^∗^	ns
*Gnaphalium supinum*	2	L^∗∗∗^	L^∗^	ns	L^∗∗^	E^†^	ns	ns	L^∗∗∗^	E^∗∗∗^
*Leucanthemopsis alpina*	2	E^†^	L^∗^	E^∗∗∗^	L^∗∗∗^	ns	E^∗∗^	L^∗^	L^∗∗∗^	L^∗^
*Polygonum viviparum*	2	ns	L^∗∗^	L^∗^	E^∗∗∗^	L^†^	L^∗∗^	E^∗∗^	–	–


*H, plant height; LA, leaf area; SLA, specific leaf area; LDMC, leaf dry matter content; LNC, leaf nitrogen content; LPC, leaf phosphorus content; N:P, N:P ratio; SN, seed number; SM, seed mass. The species were sampled at two to four sites depending on their occurrence. Capital letters indicate significantly higher values in early (E) or late (L) snowmelt areas. Significance levels are: (^∗∗∗^) P < 0.001; (^∗∗^) P < 0.01; (^∗^) P < 0.05; (^†^) marginally significant, P < 0.1; ns = not significant. SN and SM were not determined for P. viviparum because this species does not form seeds.*

### Study Design and Trait Data

**FIGURE 2:**
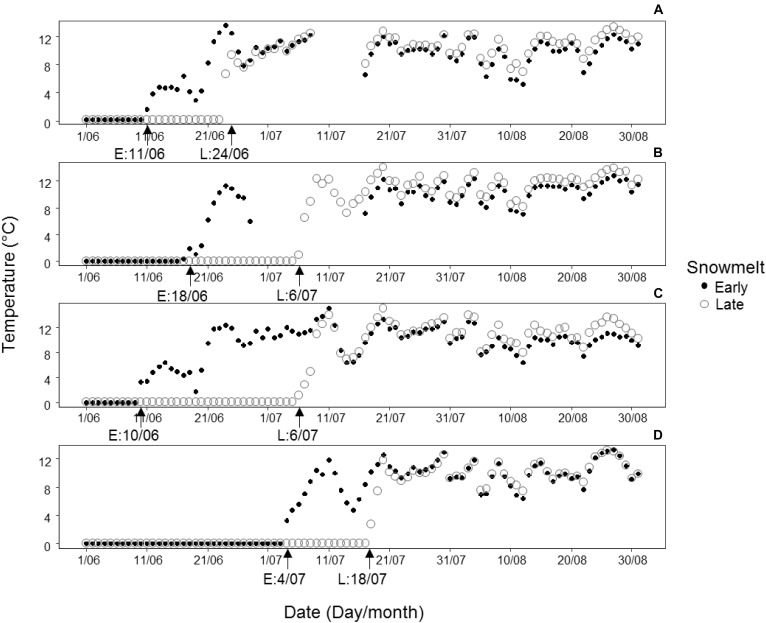
Patterns of daily mean soil temperatures (°C) registered at 5 cm soil depth during the growing season from melt-out date until the end of August in 2016 in areas of early (E, black points) and late (L, circles) snowmelt at each of the four study sites: **(A)** Forcella Travenanzes, **(B)** Pale di San Martino, **(C)** Val Martello, **(D)** Passo Gavia. The x axis represents the timescale in intervals of 10 days; the melt-out dates for the areas of early (E) and late (L) snowmelt are indicated by arrows on the x axis. For Forcella Travenanzes data from 8.07 to 16.07 are lacking because of loggers’ malfunction. For the same reason for Pale di San Martino data are lacking from 29.06 to 15.07 for early snowmelt area.

At each of the four sites, one snowbed of early and one of late snowmelt (henceforth called early snowmelt area and late snowmelt area) were chosen resulting in four early snowbeds and four late snowbeds in total (detailed coordinates and location of each snowmelt area are reported in Supplementary Table S2). The early snowmelt areas represented areas of advanced snowmelt after the above-average warm winter of 2015/2016 characterized by a low amount of snowfall (Colucci et al., 2017, see Supplementary Figure S2 for inter-annual variability of temperature and precipitation obtained from climatic stations close to the study sites). The identification of the snowmelt areas was based on previous monitoring of snowmelt timing (Carbognani et al., 2016, personal observations). The shape of a snowbed area (15–25 m^2^) was consistent with snowmelt isolines that followed the microtopographic contour lines of the snowbed. We checked the precise snowmelt timing and hence the magnitude of snow-cover duration by continuously recording soil temperatures by data loggers. A data logger (Hobo, Onset Computer Corporation, Bourne, MA, United States) was placed in the ground at 5 cm depth in each area at each site. Soil temperatures were recorded at 1 h resolution starting before the first snowfall of 2015 until end August 2016. We compared the daily mean temperatures for a period of 6 weeks, starting from the date of snow-free conditions onwards, in early vs. late snowmelt areas across sites. This period corresponded to the growing season during which all vegetative and reproductive traits reached completion. Details on snowmelt timing and temperature at each site are reported in Figure 2.

In total, seven vegetative and two reproductive traits were investigated in the selected seven snowbed species. The following vegetative key traits were determined in 30 randomly chosen individuals per snowbed area for each species: plant height (H), leaf area (LA), specific leaf area (SLA), leaf dry matter content (LDMC), leaf nitrogen content (LNC), leaf phosphorus content (LPC) and leaf N:P ratio. All of these traits are related to plants’ ecological strategies (for details see Supplementary Table S3), furthermore H and LA are also informative about competitive ability and plant persistence (Westoby, 1998; Díaz et al., 2016). SLA and LDMC are related to potential growth rate (Poorter and Remkes, 1990) while nutrient traits (LNC, LPC and N:P) can be considered as proxies of the soil nutrient status that can also assess the type of nutrient limitation (Güsewell, 2004; Pérez-Harguindeguy et al., 2013).

The two reproductive traits, number of seeds (SN) and seed mass (SM), were determined in 30 randomly chosen individuals per snowbed area for each species except *P. viviparum* which reproduces in a vegetative manner by bulbils. These traits are good indicators of the capacity of species to disperse and establish (Pierce et al., 2014; Rosbakh et al., 2014). More information about the functional significance of all of the assessed traits are provided in Supplementary Table S3.

The snowbed species have a different capacity of clonal growth. Five species, namely *G. supinum, L. alpina, P. alpina, P. viviparum*, and *V. alpina*, have very limited clonal growth (scale of few millimeters per year) using epigeous rhizomes, or in case of *V. alpina* hypogeous rhizomes (Klimešová et al., 2017)^1^. Thus, for these species, the 30 sampled plants in each snowbed area most likely represent distinct individuals. The two willow species, *S. herbacea* and *S. retusa*, have more extended clonal growth (scale of some centimeters per year) using the stem organ (Klimešová et al., 2017)^1^. For these species, we cannot exclude the possibility that some of the 30 sampled plants of a species in each snowbed area are ramets belonging to the same genet.

### Data Collection

Sampling was carried out in 2016. We surveyed the four sites several times during the growing season and sampled plants for determining vegetative traits at the peak of the growing season, i.e., on different dates at each snowbed area. Before sampling, a preliminary survey for detecting herbivore damage was done. No visible signs of damage were found, except for few individuals of *S. herbacea* at the Val Martello site. According to the protocol (Pérez-Harguindeguy et al., 2013), the damaged leaves were not sampled. Since the two *Salix* spp. are dioecious species, to avoid biases due to sexual differences in functional traits, we always sampled female individuals of *S. herbacea* and *S. retusa* in every site. This was with the exception of *S. herbacea* growing in the early snowmelt area at Forcella Travenanzes, where there were no females. For each of the 30 randomly chosen healthy individuals (ramets for *S. retusa* and *S. herbacea*) of each snowbed area, the height was measured in the field using a caliper (Metrica, precision 0.05 mm) because of the small size of the plants. SLA and LDMC were determined on the same individuals used for measuring height. To this end, the plants were harvested and immediately sealed in a plastic bag wrapped with wet paper (Pérez-Harguindeguy et al., 2013).

We let the individuals rehydrate overnight and the day after we determined fresh weight and area of one fully expanded sound leaf for individual (CanoScan LiDE 120, Canon). The same leaves were weighed again after being dried at 70°C for 72 h (Pérez-Harguindeguy et al., 2013). To determine nutrient contents, several individuals were pooled randomly to obtain enough material for chemical analyses of five replicate samples. The material was oven-dried, grinded and digested at 420°C in 3 mL of selenous H_2_SO_4_. The digests were then processed through a continuous flow autoanalyzer (FlowSys; Systea, Anagni, Italy) to determine total N and P concentration by the salicylate method and molybdenum blue method, respectively.

Seeds were collected from another set of 30 randomly selected individuals per species per snowbed area. Seed collection was performed at the time of seed maturity before dispersal. The seeds were air-dried for 30 days and then counted to determine the total number of seeds per individual for *G. supinum, L. alpina*, and *P. alpina*, and the seed number per fruit for *S. retusa, S. herbacea*, and *V. alpina*. After counting, we determined seed mass using a balance with 0.001 mg accuracy.

It was not possible to collect seeds of *P. alpina* at Pale di San Martino because at this site almost all individuals of *P. alpina* belonged to the *vivipara* variety. Neither could seeds from the small population of *S. herbacea* at Forcella Travenanzes be sampled because there were no females in that population.

As another exception, slightly fewer numbers of individuals were sampled for reproductive traits in case of one site regarding *L. alpina* [Val Martello; early (*n* = 27) and late (*n* = 26)] and *S. retusa* [Pale di San Martino; early (*n* = 19)] as these areas harbored no further individuals.

### Data Analysis

Differences in mean daily soil temperature during the growing season between early and late snowmelt areas were statistically assessed by a linear mixed effect model. We used temperature as the dependent variable, snowmelt (factor level *k* = 2: early or late) as fixed variable and snowbed site (factor level *k* = 4) as random variable.

Differences in functional trait attribute values between early and late snowmelt areas were assessed separately for each functional trait in each of the seven species. To this end, linear mixed effect models were applied using the functional trait value as the dependent variable, snowmelt (factor level *k* = 2: early or late) as fixed variable and snowbed site (factor level *k* = 2–4, depending on species occurrence in each site) as random variable. This allowed understanding how strongly each trait responded to snowmelt timing independent of the specific effects of the snowbed site.

In addition, to make the specific effect of snowbed sites apparent, linear models with each single functional trait as dependent variable and snowmelt and snowbed site as independent variables were applied. Subsequently, two-way ANOVAs were used to test for differences in functional trait values between snowbed sites and snowmelt timing.

The data were log-transformed prior to analysis whenever they did not meet the assumption of normality as assessed by the Shapiro test. All statistical analyses were performed in R (R Development Core Team, 2014 version 3.1.2). Linear mixed effect models were performed using the *nlme* package (Pinheiro et al., 2017).

## Results

### Snowmelt Timing and Soil Temperature

**FIGURE 3:**
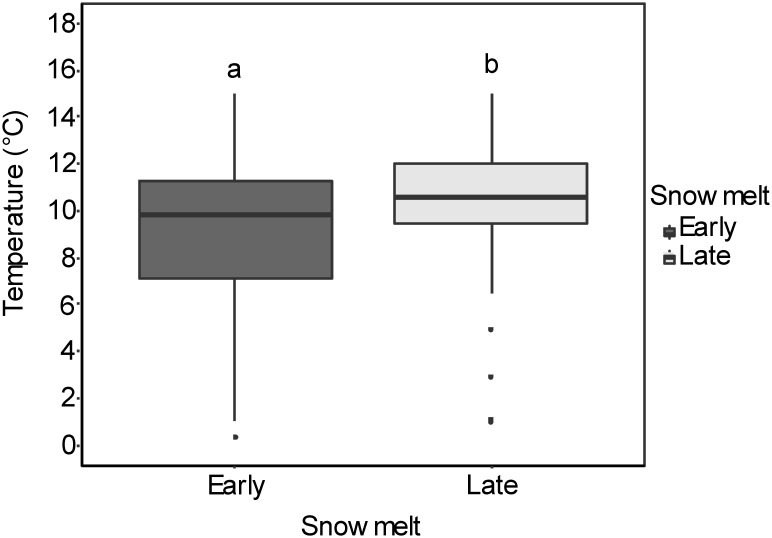
Box-whisker plots for soil temperatures in early and late snowmelt areas (number of sites = 4) for 6 weeks from melt-out date in 2016. The boxes comprise the 25th and 75th percentile with the upper and lower whiskers extending from the hinge to the upper and lower 1.5 inter-quartile ranges, respectively. The different letters indicate significant differences (*P* < 0.05) between early and late snowmelt areas. Detailed results of linear mixed effect models are given in Supplementary Table S4.

On average, snow-free conditions occurred 18 days earlier in the early snowmelt areas than in the late snowmelt areas (see Figure 2 for detailed information about snowmelt dates in each site). Daily mean soil temperature in the growing season was significantly lower in the early snowmelt areas (Figure 3).

### Vegetative Traits

**FIGURE 4:**
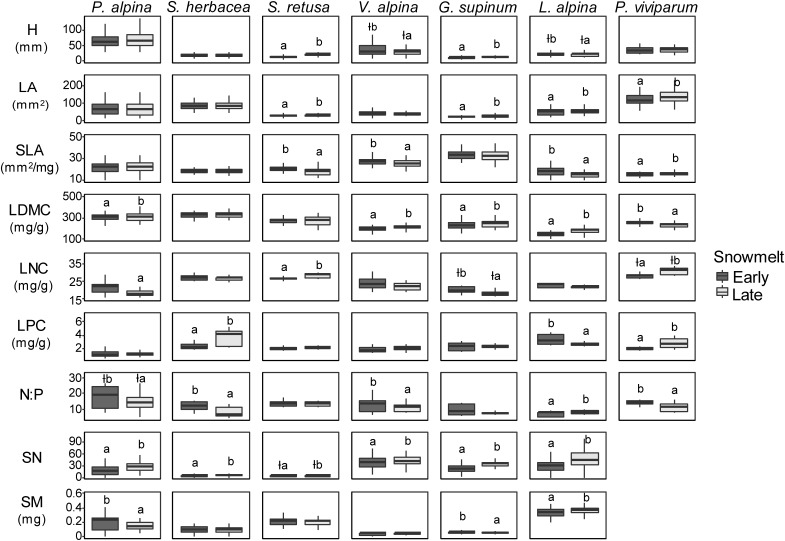
Box-whisker plots for plant traits in the seven snowbed species at early (dark gray boxes on the left-hand side of each panel) and late (light gray boxes on the right-hand side of each panel) snowmelt areas. H, plant height; LA, leaf area; SLA, specific leaf area; LDMC, leaf dry matter content; LNC, leaf nitrogen content; LPC, leaf phosphorus content; N:P, N:P ratio; SN, seed number; SM, seed mass. The species were sampled at two to four sites depending on their occurrence. *P. alpina* and *S. herbacea* were sampled for SN and SM at three and two sites, respectively, because they did not produce seeds at the other sites. The boxes comprise the 25th and 75th percentile with the upper and lower whiskers extending from the hinge to the upper and lower 1.5 inter-quartile ranges, respectively. Different letters indicate significant differences (*P* < 0.05) between early and late snowmelt areas. The symbol ł before the letters indicates a marginal significance (*P* < 0.10). See Supplementary Table S5 for values of median and first and third quartiles of early and late snowmelt groups for each species and each trait. Detailed results of linear mixed effect models are given in Supplementary Table S6.

The height of *S. retusa* and *G. supinum* was significantly lower in early snowmelt areas compared with late snowmelt areas (Figure 4 and Table 1). LA was lower in early snowmelt areas for *S. retusa, G. supinum, L. alpina*, and *P. viviparum*. SLA was higher in early snowmelt areas for *S. retusa, V. alpina*, and *L. alpina* (Figure 4 and Table 1). LDMC showed a reverse pattern compared with SLA with lower values in early snowmelt areas for *V. alpina* and *L. alpina*. *S. retusa* did not exhibit significant differences for these traits. *Poa alpina* and *G. supinum* also had lower LDMC in early snowmelt areas. Only *P. viviparum* did exhibit lower SLA and higher LDMC in early snowmelt areas (Figure 4 and Table 1). LNC in the seven species varied rather randomly in relation to snowmelt timing. While *P. alpina* presented higher LNC in early snowmelt areas, the reverse was true for *S. retusa* (Figure 4 and Table 1). LPC was lower in early snowmelt areas for *S. herbacea* and *P. viviparum* but higher in early snowmelt areas for *L. alpina* (Figure 4 and Table 1). The N:P ratio was higher in early snowmelt areas for most species: *S. herbacea, V. alpina, P. viviparum* and, marginally, for *P. alpina*. Only for *L. alpina* the N:P ratio was lower in early snowmelt areas (Figure 4 and Tables 1, 2).

**Table 2:** Mean (±SD) N:P ratio for the seven species investigated in the early and late snowmelt areas across the four study sites.

		N:P ratio	
		
Species	N. of sites	Early snowmelt	Late snowmelt
*Poa alpina*^†^	4	19.4 ± 7.9 a	15.2 ± 5.9 b
*Salix herbacea^∗∗∗^*	3	12.3 ± 2.2 a	8.2 ± 2.9 b
*Salix retusa* n.s.	2	13.7 ± 1.8	13.5 ± 1.4
*Veronica alpina^∗^*	3	13.3 ± 4.2 a	11.5 ± 2.7 b
*Gnaphalium supinum* n.s.	2	9.8 ± 3.6	8 ± 0.9
*Leucanthemopsis alpina^∗^*	2	7 ± 1.7 b	8.6 ± 1.2 a
*Polygonum viviparum^∗∗^*	2	14.4 ± 1.4 a	11.7 ± 2.8 b


*The species were sampled at two to four sites depending on their occurrence. Significance levels are (^∗∗∗^) P < 0.001; (^∗∗^) P < 0.01; (^∗^) P < 0.05; (^†^) marginally significant, P < 0.1; n.s. not significant. Different lower case letters indicate significant or marginally significant (P < 0.10) differences between early and late snowmelt areas.*

Results of additional analyses of the same data based on linear models made the effect of sites apparent. In fact, results on trait-responses of species to advanced snowmelt held true also when the effect of the snowbed sites was added (see Supplementary Table S7).

### Reproductive Traits

All seed-producing species produced less seeds in early snowmelt areas (Figure 4 and Table 1), with seed number being 40% lower for *L. alpina*, 35% lower for *P. alpina*, for *G. supinum*, 26% lower for *S. herbacea* and 13% lower for *V. alpina* in early vs. late snowmelt areas; in *S. retusa* the difference was only marginally significant (Figure 4 and Table 1). *Poa alpina* and *G. supinum* presented heavier seeds in early snowmelt areas (Figure 4 and Table 1). Conversely, *L. alpina* had lighter seeds in early snowmelt areas (Figure 4 and Table 1).

## Discussion

Our findings demonstrate that advanced snowmelt triggers the adjustment of key functional traits related to reproduction and growth of snowbed species. In particular, seed number was reduced whereas SLA increased and LDMC and LA decreased under advanced snowmelt. As at least half of the seed-producing species exhibited the same direction in the intraspecific response of these four traits (SLA, LDMC, LA, seed number), while the other species kept the traits stable, we conclude that there was a robust trend in these phenotypic trait adjustments.

Another common trend was seen in an increase in the N:P ratio under advanced snowmelt. The other traits (height, LNC, LPC, SM) varied in opposing ways, meaning that they were not indicative of a straightforward plant trait-snowmelt response across species.

### Reproductive Traits

Reduced seed production under advanced snowmelt probably depended on lower temperatures throughout the growing season in areas experiencing earlier emergence from snow. In fact, the soil temperatures during the vegetation period were significantly lower in the advanced snowmelt areas compared to the late snowmelt areas. It has already been demonstrated for another arctic-alpine species occurring at similar altitudes, i.e., *Silene acaulis*, that lowering the soil temperature by only 1°C during the maturation of flowers significantly reduced pollen tube length (Delph et al., 1997). Therefore, the temperature difference of more than 1°C in almost all of the early snowmelt areas (Forcella Travenanzes = 1.33°, Pale di San Martino = 1.35°, Val Martello = 2.01°, and Passo Gavia = 0.5°), could induce pollen limitation by reduced pollen tube growth and/or constrained pollen germination which will eventually result in lower fertilization success (Totland, 2001). A recent review confirms the importance of temperature on pollen performance and thus on seed quantity (Rosbakh et al., 2018). Reduced seed production by snowbed species under advanced snowmelt points toward negative effects on the seed rain. Fewer seeds would reduce the propagule pressure driving the establishment of new individuals and the colonization of novel sites (Lockwood et al., 2005). The need to establish on novel sites is a likely scenario under the effects of climate warming on high-altitude ecosystems (Hülber et al., 2011; Pickering et al., 2014; Komac et al., 2015). Grytnes et al. (2014) showed that novel snowbed sites at higher altitudes became available by the melting of ice and snow as a result of climate warming.

Contrary to seed number, seed mass varied little and not consistently in relation to snowmelt timing. Seed mass generally is a quite stable trait (Wellstein et al., 2013). Therefore, populations of alpine species growing over short distances are unlikely to produce seeds differing significantly in their mass (Pluess et al., 2005). In combination, less seeds without increased weight indicate less resources investment in sexual reproduction under advanced snowmelt.

### Vegetative Traits

Snowbed species tended to reduce their LA under advanced snowmelt, as already found also by Domènech et al. (2016). At the same time, our results showed higher SLA and lower LDMC under advanced snowmelt for most of the seed-producing species. Because SLA is the ratio of LA to leaf dry weight, the decrease of LA under advanced snowmelt indicates less investment in leaf structural elements, as testified also by lower LDMC. As increased SLA and decreased LDMC are both positively related to relative growth rate (Poorter and Remkes, 1990; Hulshof et al., 2013), our findings indicate a likely positive short-term effect of longer growing season on growth of seed-producing snowbed species. Enhanced growth potential and reduced resource investment in leaf structural elements under advanced snowmelt weakens snowbed species in facing the risks associated with more frequent frost events earlier in the season (Inouye, 2008; Baptist et al., 2010; Wheeler et al., 2015). Whereas species adapted to snow-free exposed habitats such as *Loiseleuria procumbens* can profit from earlier start of the growing season (Wipf et al., 2009), snowbed species appear more prone to be damaged by frost events (Bannister et al., 2005; Wheeler et al., 2014). Our temperature records did not show occurrence of frost events at the study sites. Though, as the data loggers were not positioned aboveground, light frost events may not have been detected. However, visual inspection of the vegetation did not show any sign of frost damage in any species at any site. Thus, the tendency of the snowbed species to increase their SLA under advanced snowmelt conditions may indicate a relaxation of snowbeds’ environmental constrains in the year of observation (Rosbakh et al., 2014). Some of the species investigated presented a more conservative behavior in terms of leaf structural variation, as they did not exhibit any trait adjustment under advanced snowmelt. This is for example the case of *G. supinum, S. herbacea* and *P. alpina* for SLA. Interestingly, also Baptist et al. (2010) and Komac et al. (2015) reported no plastic responses to differential snowmelt timing in terms of SLA for *G. supinum* and *P. alpina*.

The LHS (leaf-height-seed) plant ecology strategy scheme (Westoby, 1998) states that resource allocation to one set of traits – i.e., L, H, or S – is only possible by deviating resources from other sets. If responding snowbed species in early snowmelt areas tend to invest more resources to leaf development, they will invest less for growth in height and/or for seed production. Interestingly, *S. retusa* grew shorter and tended to produce less seeds while increasing SLA under advanced snowmelt.

*Polygonum viviparum*, the only non-seed-producing species, showed an opposite direction of SLA and LDMC response in relation to snow-cover duration. This probably depended on the fact that *P. viviparum* was the only species reproducing in a vegetative manner through bulbils. Previous studies showed that the amount of resources stored in bulbils is significantly lower than that stored in seeds (Ceplitis and Bengtsson, 2004; Schnittler et al., 2009). Hence, the resources saved thanks to pseudo-viviparous reproduction could be used to improve leaf development also in the case of the short growing season.

Leaf nutrient contents varied overall little in relation to snowmelt timing while the N:P ratio varied more, both in relation to species identity, and snowmelt timing (Table 2). Leaf N:P ratio has often been used for assessing nutrient limitation in different ecosystems, with N:P ratio < 10 indicating N limitation and N:P ratio >20 indicating P limitation (Güsewell, 2004). As the leaf N:P ratio varies strongly because of intrinsic differences in foliar nutrient status among species and/or plant functional types (Gerdol et al., 2017), it does not seem appropriate to define absolute thresholds of N:P ratio to assess nutrient limitation. The overall low N:P ratios found in the present study suggest a general N-limitation or N-P co-limitation in snowbed habitats, in line with previous findings (Petraglia et al., 2013, 2014b). More interestingly, the leaf N:P ratio in most species was higher under advanced snowmelt. Koller and Phoenix (2017) observed soil P mineralization rates to be less responsive to environmental change than soil N mineralization rates. So, higher N:P ratios under advanced snowmelt may reflect stronger stimulation of N mineralization by shorter snow-cover duration.

### Data Limitation

Our results are based on samples collected during the summer of 2016 after a particularly warm winter reflecting predicted magnitudes of climate warming (Supplementary Figure S2). However, even though there is a certain climatic variability between years, advanced snowmelt timing is predicted to be the norm in the future (ACIA, 2005; Pachauri et al., 2014). Regarding the snowbed species, they can be abundant within a snowbed but based on their distribution they do not always occur in every snowbed site (Pignatti and Pignatti, 2014). Therefore, four of the seven investigated species were present only in two sites. However, they showed the same trends detected for SLA, LDMC and seed number as the more replicated species.

Finally, this study investigated explicitly the phenotypic variation of the traits, without digging into the heritable component of intraspecific variability. Based on our findings of directed intraspecific functional trait adjustments, the present study opens the way to new interesting hypotheses, such as genetic differences and resulting ecotype differentiation in snowbed plant species.

## Conclusion

Taken together, our findings on foliar trait adjustments suggest improved growth conditions for snowbed species under advanced snowmelt in a warmer climatic scenario. Therefore, advanced snowmelt can improve snowbed species’ growth potential in the short term. Though, in the long term it has been predicted that ongoing improvement of growth conditions under continued future climate warming would increase the competition among plants, leading to species replacement and consequent changes in plant community composition (Körner, 2003; Cannone et al., 2007; Gottfried et al., 2012; Rosbakh et al., 2017; Winkler et al., 2018). Moreover, lower seed production can negatively affect the seed rain. A reduction of propagule pressure can be crucial in a context of loss of the present snowbed sites and the formation of new ones at higher altitudes along with climate warming. However, hypotheses on long-term effects of climate change have always to be carefully formulated, given the possibility of unexpected effects of climate conditions acting in unpredictable ways on plant population dynamics (Cannone et al., 2007). Finally, our findings encourage using plant functional traits at the intraspecific level across species as a tool to understand the future ecological challenges of plants in changing environments.

## Data Availability

All datasets generated for this study are included in the manuscript and/or the Supplementary Files.

## Author Contributions

CW, RG, MT, and RT designed the study. RT collected the data of all sites, while AP and MC contributed to data collection at the Passo Gavia site. RT and CW analyzed the data and interpreted the results. RT wrote the first draft. All authors contributed to revisions.

## Conflict of Interest Statement

The authors declare that the research was conducted in the absence of any commercial or financial relationships that could be construed as a potential conflict of interest.

## Footnotes

1 http://clopla.butbn.cas.cz/
