# Supplementary material for: Intraspecific Functional Trait Response to Advanced Snowmelt Suggests Increase of Growth Potential but Decrease of Seed Production in Snowbed Plant Species
Source: Front Plant Sci. 2019 Mar 14;10:289. doi: 10.3389/fpls.2019.00289 (PMC6426784; doi:10.3389/fpls.2019.00289)
Supplement: Supplementary file 1 [file Data_Sheet_1.docx]

Supplementary Material

**Intraspecific functional trait response to advanced snowmelt suggests increase of growth potential but decrease of seed production in snowbed plant species**

**Rita Tonin^1^, Renato Gerdol^2^, Marcello Tomaselli^3^, Alessandro Petraglia^3^, Michele Carbognani^3^, Camilla Wellstein^1*^**

*** Correspondence:** Dr. Camilla Wellstein: camilla.wellstein@unibz.it

**Supplementary tables and figures**

Table S1. Information of the occurrence and trait assessment for the seven species investigated across the four study sites. At each site, the data were collected both in early and late snowmelt areas. Assessed plant functional traits: H = plant height, SLA = specific leaf area, LDMC = leaf dry matter content, LNC = leaf nitrogen content, LPC = leaf phosphorous content, N:P = N and P ratio in the leaf, SN= seed number, SM = seed mass. Dash (-) indicates absence of the respective species in a study site.

|  | **Site** | | | |
| --- | --- | --- | --- | --- |
| **Species** | **Forcella Travenanzes** | **Pale di San Martino** | **Val Martello** | **Gavia Pass** |
|  |  |  |  |  |
| *Poa alpina* L. | H-SLA-LDMC-LNC-LPC-N:P-SN-SM | H-SLA-LDMC-LNC-LPC-N:P | H-SLA-LDMC-LNC-LPC-N:P-SN-SM | H-SLA-LDM-LNC-LPC-N:P-SN-SM |
|  |  |  |  |  |
| *Salix herbacea* L. | H-SLA-LDMC-LNC-LPC-N:P | - | H-SLA-LDMC-LNC-LPC-N:P-SN-SM | H-SLA-LDMC-LNC-LPC-N:P-SN-SM |
|  |  |  |  |  |
| *Salix retusa* L. | H-SLA-LDMC-LNC-LPC-N:P-SN-SM | H-SLA-LDMC-LNC-LPC-N:P-SN-SM | - | - |
|  |  |  |  |  |
| *Veronica alpina* L. | - | H-SLA-LDMC-LNC-LPC-N:P-SN-SM | H-SLA-LDMC-LNC-LPC-N:P-SN-SM | H-SLA-LDMC-LNC-LPC-N:P-SN-SM |
|  |  |  |  |  |
| *Gnaphalium supinum* L. | - | - | H-SLA-LDMC-LNC-LPC-N:P-SN-SM | H-SLA-LDMC-LNC-LPC-N:P-SN-SM |
|  |  |  |  |  |
| *Leucanthemopsis alpina* (L.) Heywood | - | - | H-SLA-LDMC-LNC-LPC-N:P-SN-SM | H-SLA-LDMC-LNC-LPC-N:P-SN-SM |
|  |  |  |  |  |
| *Polygonum viviparum* L. | H-SLA-LDMC-LNC-LPC-N:P | H-SLA-LDM-LNC-LPC-N:P | - | - |
|  |  |  |  |  |

Table S2. Detailed coordinates of early and late snowmelt areas within each site (FT = Forcella Travenanzes, PSM = Pale di San Martino, VM = Val Martello, GV = Passo Gavia).

|  |  | Coordinates | |
| --- | --- | --- | --- |
| Site | Snowmelt area | Latitude | Longitude |
| FT | Early | 46°31'53.4'' N | 12°1'16.4''E |
|  | Late | 46°31'55.6" N | 12°1'20.6'' E |
| PSM | Early | 46°16'0.7'' N | 11°50'25.2'' E |
|  | Late | 46°16'4.7'' N | 11°50'29.2'' E |
| VM | Early | 46°28'5'' N | 10°40'19.4'' E |
|  | Late | 46°28'0.2'' N | 10°40'17.2'' E |
| GV | Early | 46°20'26.1'' N | 10°29'57.4'' E |
|  | Late | 46°20'25.3'' N | 10°29'56.8'' E |

Table S3. Investigated plant functional traits, their abbreviations, definitions, units of measurement, functional significance and respective literature reference, modified from Wellstein et al. (2013)

| **Trait** | **Abbreviation** | **Description** | **Units** | **Functional Meaning** | **Literature** |
| --- | --- | --- | --- | --- | --- |
| *Vegetative traits* |  |  |  |  |  |
| **Plant height** | H | Distance between the highest photosynthetic tissue and the base of the plant | mm | Associated with competitive ability, reproductive size, whole plant fecundity and generation time after disturbance. Positively related to seed dispersal capacity. | Kleyer et al. (2008); Pérez-Harguindeguy et al. (2013); Wellstein et al. (2013) |
|  |  |  |  |  |  |
|  |  |  |  |  |  |
| **Leaf area** | LA | One-sided area of an individual leaf | mm^2^ | Linked to allometric factors, competitive ability and plant persistence | Pérez-Harguindeguy et al. (2013); Díaz et al. (2016) |
| **Specific leaf area** | SLA | Ratio of fresh leaf area to leaf dry mass | mm^2^/mg | Positively correlated with potential relative growth rate (RGR) and mass-based light-saturated photosynthetic rate. Scales negatively with leaf longevity and C investment in secondary structural compounds. Plasticity of SLA well documented. | Kleyer et al. (2008); Pérez-Harguindeguy et al. (2013); Wellstein et al. (2013); Westoby (1998) |
|  |  |  |  |  |  |
|  |  |  |  |  |  |
|  |  |  |  |  |  |
| **Leaf dry-matter content** | LDMC | Ratio of dry leaf mass to fresh leaf mass | mg/g | Positively related to leaf lifespan and structural investment while negatively with potential RGR. Scales with the inverse of SLA. Leaves with high LDMC values tend to have a low turn-over enhancing nutrients conservation. | Kleyer et al. (2008); Pérez-Harguindeguy et al. (2013); Wellstein et al. (2013) |
|  |  |  |  |  |  |
|  |  |  |  |  |  |
|  |  |  |  |  |  |
| **Leaf nitrogen content** | LNC | Total amount of N per unit of dry leaf mass | mg/g | High LNC or LPC values are generally associated with high nutritional quality to the consumers in the food web. These traits vary significantly with environmental N and P availability. | Pérez-Harguindeguy et al. (2013) |
|  |  |  |  |  |  |
| **Leaf phosphorus content** | LPC | Total amount of P per unit of dry leaf mass | mg/g |  | Pérez-Harguindeguy et al. (2013) |
|  |  |  |  |  |  |
| **N:P ratio** | N:P | Ratio between LNC and LPC | - | Commonly used to assess the type of nutrient limitation. Optimal values of N:P ratio depend on species, growth rate, plant age and plant parts. Low N:P ratios indicate N-limitation while some authors suggest that high N:P rations indicate P-limitation alone. | Güsewell (2004); Güsewell and Koerselman (2002) |
|  |  |  |  |  |  |
|  |  |  |  |  |  |

Table S3. continued

| **Trait** | **Abbreviation** | **Description** | **Units** | **Functional Meaning** | **Literature** |
| --- | --- | --- | --- | --- | --- |
| *Reproductive traits* |  |  |  |  |  |
| **Seed Number** | SN | Total seed production per ramet/shoot of a species | - | Associated with plant size and dispersal capacity. Because the existence of seed size/number trade-offs, it can be used in association with seed mass to understand plants' reproductive strategies. | Kleyer et al. (2008); Leishman (2001) |
|  |  |  |  |  |  |
|  |  |  |  |  |  |
| **Seed Mass** | SM | Air dried weight of seeds | mg | In the light of seed size/number trade-offs, it has been demonstrated that larger seeds confer more chances for seedling survival and establishment especially under shaded conditions. Relation to seed dispersion and persistence in the soil. | Kleyer et al. (2008); Pérez- Harguindeguy et al. (2013); Wellstein et al. (2013); Leishman (2001); Venable and Brown (1988) |
|  |  |  |  |  |  |
|  |  |  |  |  |  |

Table S4. Results of linear mixed effect models for the daily mean temperature in early and late snowbeds across four study areas. The daily mean temperature was analyzed as the response variable, snowmelt as the fixed variable, and the site as the random variable. Early snowmelt area was considered as the base-line level.

| **Fixed effect** | **Value** | **Std.Error** | **DF** | **t-value** | **p-value** |
| --- | --- | --- | --- | --- | --- |
| (Intercept) | 8.966764 | 0.2133418 | 313 | 42.03003 | 0 |
| Snowmelt | 1.493532 | 0.2900851 | 313 | 5.14860 | 0 |

Table S5. Median and first and third quartiles of early (E) and late (L) snowmelt groups for each trait for the species (A) *Poa alpina*, (B) *Salix herbacea*, (C) *Salix retusa*, (D) *Veronica alpina*, (E) *Gnaphalium supinum*, (F) *Leucanthemopsis alpina*, (G) *Polygonum viviparum*.

| **A** | **Species** | **Trait** | **Snowmelt** | **First quartile** | **Median** | **Third quartile** |
| --- | --- | --- | --- | --- | --- | --- |
|  | *P. alpina* | H | E | 52.00 | 66.00 | 95.75 |
|  |  |  | L | 52.75 | 70.00 | 97.75 |
|  |  | LA | E | 36.33 | 60.65 | 95.40 |
|  |  |  | L | 35.58 | 63.35 | 92.30 |
|  |  | SLA | E | 17.58 | 21.20 | 24.66 |
|  |  |  | L | 17.69 | 21.65 | 25.31 |
|  |  | LDMC | E | 287.02 | 304.59 | 326.16 |
|  |  |  | L | 277.37 | 312.61 | 338.89 |
|  |  | LNC | E | 17.51 | 20.68 | 22.89 |
|  |  |  | L | 13.57 | 14.87 | 18.16 |
|  |  | LPC | E | 0.81 | 1.07 | 1.50 |
|  |  |  | L | 0.91 | 1.09 | 1.27 |
|  |  | N:P | E | 10.88 | 20.59 | 25.46 |
|  |  |  | L | 11.35 | 14.38 | 17.92 |
|  |  | SN | E | 8.50 | 16.00 | 26.00 |
|  |  |  | L | 17.00 | 25.00 | 35.50 |
|  |  | SM | E | 0.07 | 0.22 | 0.27 |
|  |  |  | L | 0.10 | 0.14 | 0.26 |

| **B** | **Species** | **Trait** | **Snowmelt** | **First quartile** | **Median** | **Third quartile** |
| --- | --- | --- | --- | --- | --- | --- |
|  | *S. herbacea* | H | E | 15.85 | 18.48 | 22.78 |
|  |  |  | L | 15.00 | 18.53 | 21.69 |
|  |  | LA | E | 70.85 | 84.30 | 99.08 |
|  |  |  | L | 68.70 | 85.75 | 99.83 |
|  |  | SLA | E | 17.19 | 18.27 | 19.40 |
|  |  |  | L | 16.91 | 18.36 | 19.36 |
|  |  | LDMC | E | 305.65 | 331.91 | 343.20 |
|  |  |  | L | 313.89 | 326.54 | 345.05 |
|  |  | LNC | E | 25.79 | 26.89 | 28.51 |
|  |  |  | L | 25.87 | 26.90 | 27.56 |
|  |  | LPC | E | 1.94 | 2.20 | 2.56 |
|  |  |  | L | 2.32 | 4.08 | 4.56 |
|  |  | N:P | E | 10.60 | 12.13 | 14.74 |
|  |  |  | L | 6.12 | 6.68 | 11.15 |
|  |  | SN | E | 1.23 | 2.83 | 4.79 |
|  |  |  | L | 3.20 | 4.38 | 5.61 |
|  |  | SM | E | 0.06 | 0.10 | 0.13 |
|  |  |  | L | 0.07 | 0.10 | 0.12 |

| **C** | **Species** | **Trait** | **Snowmelt** | **First quartile** | **Median** | **Third quartile** |
| --- | --- | --- | --- | --- | --- | --- |
|  | *S.retusa* | H | E | 11.26 | 13.00 | 16.33 |
|  |  |  | L | 17.70 | 20.85 | 23.25 |
|  |  | LA | E | 22.95 | 25.85 | 30.48 |
|  |  |  | L | 27.10 | 30.15 | 34.88 |
|  |  | SLA | E | 19.11 | 20.34 | 22.11 |
|  |  |  | L | 15.18 | 17.66 | 20.18 |
|  |  | LDMC | E | 256.29 | 272.12 | 285.34 |
|  |  |  | L | 238.55 | 275.13 | 304.77 |
|  |  | LNC | E | 26.06 | 26.56 | 27.28 |
|  |  |  | L | 27.49 | 28.90 | 29.66 |
|  |  | LPC | E | 1.80 | 1.93 | 2.15 |
|  |  |  | L | 1.93 | 2.20 | 2.28 |
|  |  | N:P | E | 12.49 | 13.47 | 14.60 |
|  |  |  | L | 12.15 | 13.52 | 14.50 |
|  |  | SN | E | 1.25 | 2.11 | 2.75 |
|  |  |  | L | 1.64 | 2.35 | 3.26 |
|  |  | SM | E | 0.17 | 0.21 | 0.24 |
|  |  |  | L | 0.17 | 0.21 | 0.23 |

| **D** | **Species** | **Trait** | **Snowmelt** | **First quartile** | **Median** | **Third quartile** |
| --- | --- | --- | --- | --- | --- | --- |
|  | *V. alpina* | H | E | 21.50 | 30.00 | 49.70 |
|  |  |  | L | 23.14 | 29.55 | 36.93 |
|  |  | LA | E | 28.40 | 38.30 | 50.60 |
|  |  |  | L | 30.50 | 37.35 | 44.20 |
|  |  | SLA | E | 24.39 | 26.67 | 29.20 |
|  |  |  | L | 22.96 | 24.67 | 27.43 |
|  |  | LDMC | E | 186.50 | 199.17 | 215.89 |
|  |  |  | L | 204.86 | 218.09 | 230.32 |
|  |  | LNC | E | 21.32 | 22.74 | 26.14 |
|  |  |  | L | 19.80 | 22.08 | 24.10 |
|  |  | LPC | E | 1.52 | 1.62 | 2.04 |
|  |  |  | L | 1.82 | 1.94 | 2.29 |
|  |  | N:P | E | 9.24 | 13.81 | 16.00 |
|  |  |  | L | 9.14 | 11.92 | 12.94 |
|  |  | SN | E | 25.50 | 37.31 | 47.99 |
|  |  |  | L | 34.13 | 41.25 | 51.50 |
|  |  | SM | E | 0.02 | 0.04 | 0.05 |
|  |  |  | L | 0.03 | 0.04 | 0.05 |

| **E** | **Species** | **Trait** | **Snowmelt** | **First quartile** | **Median** | **Third quartile** |
| --- | --- | --- | --- | --- | --- | --- |
|  | *G. supinum* | H | E | 8.76 | 10.13 | 12.60 |
|  |  |  | L | 10.59 | 12.65 | 14.80 |
|  |  | LA | E | 16.43 | 19.15 | 23.20 |
|  |  |  | L | 17.93 | 23.85 | 30.63 |
|  |  | SLA | E | 30.23 | 32.86 | 35.89 |
|  |  |  | L | 28.81 | 32.30 | 35.74 |
|  |  | LDMC | E | 205.35 | 226.54 | 254.21 |
|  |  |  | L | 220.77 | 243.70 | 266.85 |
|  |  | LNC | E | 18.83 | 19.48 | 21.91 |
|  |  |  | L | 16.95 | 18.13 | 19.35 |
|  |  | LPC | E | 1.64 | 2.23 | 2.82 |
|  |  |  | L | 2.15 | 2.31 | 2.50 |
|  |  | N:P | E | 6.55 | 9.31 | 13.39 |
|  |  |  | L | 7.45 | 7.73 | 8.39 |
|  |  | SN | E | 15.00 | 22.50 | 28.25 |
|  |  |  | L | 29.00 | 34.00 | 37.25 |
|  |  | SM | E | 0.05 | 0.06 | 0.07 |
|  |  |  | L | 0.04 | 0.05 | 0.06 |

| **F** | **Species** | **Trait** | **Snowmelt** | **First quartile** | **Median** | **Third quartile** |
| --- | --- | --- | --- | --- | --- | --- |
|  | *L. alpina* | H | E | 19.56 | 23.00 | 26.13 |
|  |  |  | L | 15.99 | 21.00 | 25.00 |
|  |  | LA | E | 30.90 | 50.70 | 61.10 |
|  |  |  | L | 44.48 | 50.30 | 66.50 |
|  |  | SLA | E | 15.30 | 17.95 | 20.90 |
|  |  |  | L | 12.62 | 14.36 | 16.32 |
|  |  | LDMC | E | 137.65 | 150.55 | 160.88 |
|  |  |  | L | 162.28 | 178.97 | 197.77 |
|  |  | LNC | E | 21.59 | 22.96 | 23.90 |
|  |  |  | L | 21.45 | 22.14 | 22.46 |
|  |  | LPC | E | 2.66 | 3.22 | 4.24 |
|  |  |  | L | 2.44 | 2.60 | 2.76 |
|  |  | N:P | E | 5.55 | 7.03 | 8.50 |
|  |  |  | L | 7.65 | 8.53 | 9.58 |
|  |  | SN | E | 17.00 | 28.00 | 37.00 |
|  |  |  | L | 32.75 | 42.50 | 61.50 |
|  |  | SM | E | 0.29 | 0.33 | 0.38 |
|  |  |  | L | 0.33 | 0.36 | 0.40 |

| **G** | **Species** | **Trait** | **Snowmelt** | **First quartile** | **Median** | **Third quartile** |
| --- | --- | --- | --- | --- | --- | --- |
|  | *P. viviparum* | H | E | 26.83 | 33.85 | 43.76 |
|  |  |  | L | 30.89 | 36.88 | 41.79 |
|  |  | LA | E | 100.85 | 113.15 | 144.05 |
|  |  |  | L | 114.58 | 133.65 | 169.28 |
|  |  | SLA | E | 13.96 | 15.08 | 16.21 |
|  |  |  | L | 14.65 | 15.48 | 17.20 |
|  |  | LDMC | E | 242.91 | 255.68 | 267.66 |
|  |  |  | L | 220.38 | 235.89 | 247.00 |
|  |  | LNC | E | 27.35 | 28.13 | 29.50 |
|  |  |  | L | 29.16 | 31.26 | 32.89 |
|  |  | LPC | E | 1.87 | 1.98 | 2.07 |
|  |  |  | L | 2.19 | 2.60 | 3.42 |
|  |  | N:P | E | 13.63 | 14.46 | 15.54 |
|  |  |  | L | 9.17 | 11.14 | 14.01 |

Table S6. Results of linear mixed effect models for each species and each trait. Each single trait was analyzed as the response variable, snowmelt as the fixed variable and site as the random variable. The basic level (intercept) corresponds to the early area.

| **Species** | **Trait** | **Fixed effect** | **Value** | **Std.Error** | **DF** | **t-value** | **p-value** |
| --- | --- | --- | --- | --- | --- | --- | --- |
| *P. alpina* | log(H) | (intercept) | 43.02 | 0.21 | 235 | 20.97 | 0.00 |
|  |  | snowmelt | -0.01 | 0.04 | 235 | -0.15 | 0.88 |
|  | log(LA) | (intercept) | 4.06 | 0.26 | 235 | 15.82 | 0.00 |
|  |  | snowmelt | 0.02 | 0.06 | 235 | 0.29 | 0.78 |
|  | SLA | (intercept) | 21.33 | 2.39 | 235 | 8.94 | 0.00 |
|  |  | snowmelt | -0.48 | 0.53 | 235 | -0.91 | 0.37 |
|  | LDMC | (intercept) | 305.05 | 13.58 | 235 | 22.46 | 0.00 |
|  |  | snowmelt | 8.58 | 3.85 | 235 | 2.23 | 0.03 |
|  | LNC | (intercept) | 19.96 | 1.38 | 35 | 14.49 | 0.00 |
|  |  | snowmelt | -4.11 | 1.10 | 35 | -3.73 | 0.00 |
|  | LPC | (intercept) | 1.19 | 0.14 | 35 | 8.29 | 0.00 |
|  |  | snowmelt | -0.04 | 0.13 | 35 | -0.30 | 0.76 |
|  | log(N:P) | (intercept) | 2.88 | 0.15 | 35 | 18.75 | 0.00 |
|  |  | snowmelt | -0.23 | 0.11 | 35 | -1.97 | 0.06 |
|  | SN | (intercept) | 17.66 | 3.13 | 176 | 5.64 | 0.00 |
|  |  | snowmelt | 10.48 | 2.04 | 176 | 5.14 | 0.00 |
|  | SM | (intercept) | 0.19 | 0.04 | 176 | 4.71 | 0.00 |
|  |  | snowmelt | -0.04 | 0.01 | 176 | -4.70 | 0.00 |
|  |  |  |  |  |  |  |  |
| *S. herbacea* | H | (intercept) | 19.21 | 1.24 | 176 | 15.49 | 0.00 |
|  |  | snowmelt | -1.06 | 0.72 | 176 | -1.48 | 0.14 |
|  | LA | (intercept) | 87.96 | 5.67 | 176 | 15.50 | 0.00 |
|  |  | snowmelt | 0.18 | 3.96 | 176 | 0.05 | 0.96 |
|  | SLA | (intercept) | 18.29 | 0.52 | 176 | 35.37 | 0.00 |
|  |  | snowmelt | 0.02 | 0.25 | 176 | 0.06 | 0.95 |
|  | LDMC | (intercept) | 326.61 | 8.43 | 176 | 38.75 | 0.00 |
|  |  | snowmelt | 0.73 | 3.65 | 176 | 0.20 | 0.84 |
|  | LNC | (intercept) | 27.28 | 0.44 | 26 | 61.37 | 0.00 |
|  |  | snowmelt | -0.27 | 0.60 | 26 | -0.45 | 0.65 |
|  | LPC | (intercept) | 2.29 | 0.49 | 26 | 4.63 | 0.00 |
|  |  | snowmelt | 1.37 | 0.19 | 26 | 7.40 | 0.00 |
|  | N:P | (intercept) | 12.30 | 1.69 | 26 | 7.27 | 0.00 |
|  |  | snowmelt | -4.10 | 0.38 | 26 | -10.72 | 0.00 |
|  | SN | (intercept) | 3.34 | 1.33 | 117 | 2.50 | 0.01 |
|  |  | snowmelt | 1.18 | 0.35 | 117 | 3.36 | 0.00 |
|  | SM | (intercept) | 0.09 | 0.02 | 117 | 5.83 | 0.00 |
|  |  | snowmelt | 0.00 | 0.01 | 117 | 0.28 | 0.78 |
|  |  |  |  |  |  |  |  |
| *S.retusa* | H | (intercept) | 13.46 | 0.58 | 117 | 23.11 | 0.00 |
|  |  | snowmelt | 7.13 | 0.82 | 117 | 8.65 | 0.00 |
|  | LA | (intercept) | 26.74 | 1.78 | 117 | 15.01 | 0.00 |
|  |  | snowmelt | 5.23 | 1.28 | 117 | 4.07 | 0.00 |
|  | SLA | (intercept) | 20.68 | 1.30 | 117 | 15.88 | 0.00 |
|  |  | snowmelt | -2.18 | 0.59 | 117 | -3.69 | 0.00 |
|  | log(LDMC) | (intercept) | 5.60 | 0.05 | 117 | 102.45 | 0.00 |
|  |  | snowmelt | 0.00 | 0.02 | 117 | -0.17 | 0.87 |
|  | LNC | (intercept) | 26.66 | 0.40 | 17 | 65.97 | 0.00 |
|  |  | snowmelt | 1.89 | 0.53 | 17 | 3.54 | 0.00 |
|  | LPC | (intercept) | 1.97 | 0.07 | 17 | 26.57 | 0.00 |
|  |  | snowmelt | 0.16 | 0.10 | 17 | 1.54 | 0.14 |
|  | N:P | (intercept) | 13.71 | 0.52 | 17 | 26.57 | 0.00 |
|  |  | snowmelt | -0.22 | 0.73 | 17 | -0.30 | 0.77 |
|  | SN | (intercept) | 0.62 | 0.08 | 105 | 7.97 | 0.00 |
|  |  | snowmelt | 0.18 | 0.10 | 105 | 1.75 | 0.08 |
|  | SM | (intercept) | 0.21 | 0.01 | 105 | 26.83 | 0.00 |
|  |  | snowmelt | 0.00 | 0.01 | 105 | -0.16 | 0.88 |
|  |  |  |  |  |  |  |  |
| *V. alpina* | log(H) | (intercept) | 3.47 | 0.24 | 176 | 14.63 | 0.00 |
|  |  | snowmelt | -0.10 | 0.05 | 176 | -1.89 | 0.06 |
|  | log(LA) | (intercept) | 3.69 | 0.13 | 176 | 29.00 | 0.00 |
|  |  | snowmelt | -0.08 | 0.05 | 176 | -1.55 | 0.12 |
|  | log(SLA) | (intercept) | 3.28 | 0.02 | 176 | 163.24 | 0.00 |
|  |  | snowmelt | -0.06 | 0.02 | 176 | -2.92 | 0.00 |
|  | LDMC | (intercept) | 200.32 | 2.26 | 176 | 88.79 | 0.00 |
|  |  | snowmelt | 17.47 | 3.17 | 176 | 5.52 | 0.00 |
|  | LNC | (intercept) | 23.22 | 0.86 | 26 | 27.13 | 0.00 |
|  |  | snowmelt | -0.97 | 1.21 | 26 | -0.80 | 0.43 |
|  | LPC | (intercept) | 1.90 | 0.31 | 26 | 6.17 | 0.00 |
|  |  | snowmelt | 0.11 | 0.13 | 26 | 0.85 | 0.40 |
|  | N:P | (intercept) | 13.27 | 1.96 | 26 | 6.77 | 0.00 |
|  |  | snowmelt | -1.73 | 0.83 | 26 | -2.09 | 0.05 |
|  | SN | (intercept) | 36.97 | 2.39 | 176 | 15.45 | 0.00 |
|  |  | snowmelt | 5.40 | 2.20 | 176 | 2.45 | 0.02 |
|  | SM | (intercept) | 0.04 | 0.01 | 176 | 6.52 | 0.00 |
|  |  | snowmelt | 0.00 | 0.00 | 176 | 1.55 | 0.12 |
|  |  |  |  |  |  |  |  |
| *G. supinum* | H | (intercept) | 10.81 | 0.56 | 117 | 19.45 | 0.00 |
|  |  | snowmelt | 2.12 | 0.64 | 117 | 3.31 | 0.00 |
|  | log(LA) | (intercept) | 2.97 | 0.05 | 117 | 59.75 | 0.00 |
|  |  | snowmelt | 0.13 | 0.06 | 117 | 2.09 | 0.04 |
|  | log(SLA) | (intercept) | 3.51 | 0.08 | 117 | 46.13 | 0.00 |
|  |  | snowmelt | -0.04 | 0.03 | 117 | -1.40 | 0.16 |
|  | LDMC | (intercept) | 229.24 | 21.75 | 117 | 10.54 | 0.00 |
|  |  | snowmelt | 17.31 | 5.25 | 117 | 3.30 | 0.00 |
|  | LNC | (intercept) | 20.07 | 0.58 | 17 | 34.46 | 0.00 |
|  |  | snowmelt | -1.69 | 0.82 | 17 | -2.05 | 0.06 |
|  | LPC | (intercept) | 2.26 | 0.41 | 17 | 5.50 | 0.00 |
|  |  | snowmelt | 0.06 | 0.14 | 17 | 0.45 | 0.66 |
|  | log(N:P) | (intercept) | 2.22 | 0.19 | 17 | 11.59 | 0.00 |
|  |  | snowmelt | -0.15 | 0.09 | 17 | -1.61 | 0.13 |
|  | SN | (intercept) | 22.47 | 4.77 | 117 | 4.71 | 0.00 |
|  |  | snowmelt | 11.42 | 1.48 | 117 | 7.70 | 0.00 |
|  | SM | (intercept) | 0.06 | 0.01 | 117 | 6.47 | 0.00 |
|  |  | snowmelt | -0.01 | 0.00 | 117 | -4.43 | 0.00 |
|  |  |  |  |  |  |  |  |
| *L. alpina* | H | (intercept) | 23.23 | 1.26 | 117 | 18.49 | 0.00 |
|  |  | snowmelt | -2.08 | 1.10 | 117 | -1.89 | 0.06 |
|  | LA | (intercept) | 48.39 | 2.56 | 117 | 18.92 | 0.00 |
|  |  | snowmelt | 8.39 | 3.62 | 117 | 2.32 | 0.02 |
|  | log(SLA) | (intercept) | 2.90 | 0.07 | 117 | 44.04 | 0.00 |
|  |  | snowmelt | -0.23 | 0.04 | 117 | -5.79 | 0.00 |
|  | LDMC | (intercept) | 149.74 | 3.54 | 117 | 42.33 | 0.00 |
|  |  | snowmelt | 31.08 | 4.25 | 117 | 7.32 | 0.00 |
|  | LNC | (intercept) | 22.73 | 0.38 | 17 | 59.16 | 0.00 |
|  |  | snowmelt | -0.64 | 0.54 | 17 | -1.18 | 0.26 |
|  | log(LPC) | (intercept) | 1.20 | 0.10 | 17 | 12.10 | 0.00 |
|  |  | snowmelt | -0.24 | 0.07 | 17 | -3.35 | 0.00 |
|  | N:P | (intercept) | 7.05 | 0.61 | 17 | 11.50 | 0.00 |
|  |  | snowmelt | 1.52 | 0.64 | 17 | 2.39 | 0.03 |
|  | SN | (intercept) | 27.47 | 7.25 | 110 | 3.79 | 0.00 |
|  |  | snowmelt | 18.16 | 3.35 | 110 | 5.42 | 0.00 |
|  | SM | (intercept) | 0.33 | 0.01 | 110 | 37.11 | 0.00 |
|  |  | snowmelt | 0.03 | 0.01 | 110 | 2.02 | 0.05 |
|  |  |  |  |  |  |  |  |
| *P. viviparum* | log(H) | (intercept) | 3.53 | 0.16 | 117 | 22.27 | 0.00 |
|  |  | snowmelt | 0.04 | 0.04 | 117 | 1.19 | 0.24 |
|  | LA | (intercept) | 122.17 | 11.93 | 117 | 10.24 | 0.00 |
|  |  | snowmelt | 19.73 | 7.43 | 117 | 2.65 | 0.01 |
|  | SLA | (intercept) | 15.08 | 0.23 | 117 | 66.50 | 0.00 |
|  |  | snowmelt | 0.85 | 0.32 | 117 | 2.65 | 0.01 |
|  | LDMC | (intercept) | 258.06 | 6.49 | 117 | 39.76 | 0.00 |
|  |  | snowmelt | -25.36 | 4.67 | 117 | -5.43 | 0.00 |
|  | LNC | (intercept) | 28.44 | 0.79 | 17 | 36.19 | 0.00 |
|  |  | snowmelt | 2.24 | 1.11 | 17 | 2.01 | 0.06 |
|  | LPC | (intercept) | 1.99 | 0.32 | 17 | 6.15 | 0.00 |
|  |  | snowmelt | 0.77 | 0.20 | 17 | 3.77 | 0.00 |
|  | N:P | (intercept) | 14.42 | 1.39 | 17 | 10.38 | 0.00 |
|  |  | snowmelt | -2.67 | 0.81 | 17 | -3.28 | 0.00 |

Table S7. Results of ANOVA on the effect of snowmelt timing and site for each species and each trait. Each single trait was analyzed as the response variable, and snowmelt and site as independent variables.

| **Species** | **Trait** | **variables** | **Df** | **Sum Sq** | **Mean Sq** | **F value** | **Pr(>F)** |
| --- | --- | --- | --- | --- | --- | --- | --- |
| *P.alpina* | log(H) | snowmelt | 1 | 0.00 | 0.00 | 0.02 | 0.88 |
|  |  | site | 3 | 29.95 | 9.98 | 86.63 | 0.00 |
|  |  | residuals | 235 | 27.09 | 0.12 |  |  |
|  | log(LA) | snowmelt | 1 | 0.02 | 0.02 | 0.08 | 0.78 |
|  |  | site | 3 | 46.84 | 15.61 | 77.21 | 0.00 |
|  |  | residuals | 235 | 47.52 | 0.20 |  |  |
|  | SLA | snowmelt | 1 | 13.80 | 13.85 | 0.82 | 0.37 |
|  |  | site | 3 | 4050.60 | 1350.22 | 79.90 | 0.00 |
|  |  | residuals | 235 | 3971.10 | 16.90 |  |  |
|  | LDMC | snowmelt | 1 | 4418 | 4418 | 4.97 | 0.03 |
|  |  | site | 3 | 130106 | 43369 | 48.75 | 0.00 |
|  |  | residuals | 235 | 209067 | 890 |  |  |
|  | LNC | snowmelt | 1 | 169.29 | 169.29 | 13.93 | 0.00 |
|  |  | site | 3 | 191.02 | 63.67 | 5.24 | 0.00 |
|  |  | residuals | 35 | 425.42 | 12.16 |  |  |
|  | LPC | snowmelt | 1 | 0.02 | 0.02 | 0.09 | 0.76 |
|  |  | site | 3 | 1.98 | 0.66 | 4.01 | 0.01 |
|  |  | residuals | 35 | 5.76 | 0.16 |  |  |
|  | log(N:P) | snowmelt | 1 | 0.51 | 0.51 | 3.86 | 0.06 |
|  |  | site | 3 | 2.43 | 0.81 | 6.12 | 0.00 |
|  |  | residuals | 35 | 4.63 | 0.13 |  |  |
|  | SN | snowmelt | 1 | 4940 | 4940 | 26.43 | 0.00 |
|  |  | site | 2 | 3159 | 1579 | 8.45 | 0.00 |
|  |  | residuals | 176 | 32896 | 187 |  |  |
|  | SM | snowmelt | 1 | 0.08 | 0.08 | 22.13 | 0.00 |
|  |  | site | 2 | 0.59 | 0.29 | 84.78 | 0.00 |
|  |  | residuals | 176 | 0.61 | 0.00 |  |  |
|  |  |  |  |  |  |  |  |
| *S. herbacea* | H | snowmelt | 1 | 50.40 | 50.40 | 2.18 | 0.14 |
|  |  | site | 2 | 507.30 | 253.64 | 10.97 | 0.00 |
|  |  | residuals | 176 | 4067.60 | 23.11 |  |  |
|  | LA | snowmelt | 1 | 1.00 | 1.50 | 0.00 | 0.96 |
|  |  | site | 2 | 10184 | 5092 | 7.23 | 0.00 |
|  |  | residuals | 176 | 123955 | 704.30 |  |  |
|  | SLA | snowmelt | 1 | 0.01 | 0.01 | 0.00 | 0.95 |
|  |  | site | 2 | 90.44 | 45.22 | 15.69 | 0.00 |
|  |  | residuals | 176 | 507.35 | 2.88 |  |  |
|  | LDMC | snowmelt | 1 | 24.00 | 24.10 | 0.04 | 0.84 |
|  |  | site | 2 | 24370.00 | 12184.80 | 20.30 | 0.00 |
|  |  | residuals | 176 | 105648 | 600.30 |  |  |
|  | LNC | snowmelt | 1 | 0.56 | 0.55 | 0.21 | 0.65 |
|  |  | site | 2 | 6.48 | 3.24 | 1.20 | 0.32 |
|  |  | residuals | 26 | 69.91 | 2.69 |  |  |
|  | LPC | snowmelt | 1 | 14.15 | 14.15 | 54.71 | 0.00 |
|  |  | site | 2 | 14.08 | 7.04 | 27.24 | 0.00 |
|  |  | residuals | 26 | 6.72 | 0.26 |  |  |
|  | N:P | snowmelt | 1 | 126.15 | 126.15 | 114.94 | 0.00 |
|  |  | site | 2 | 169.54 | 84.77 | 77.24 | 0.00 |
|  |  | residuals | 26 | 28.54 | 1.10 |  |  |
|  | SN | snowmelt | 1 | 42.01 | 42.01 | 11.27 | 0.00 |
|  |  | site | 1 | 209.67 | 209.67 | 56.26 | 0.00 |
|  |  | residuals | 117 | 436.01 | 3.73 |  |  |
|  | SM | snowmelt | 1 | 0.07 | 0.01 | 10.20 | 0.00 |
|  |  | site | 1 | 0.00 | 0.01 | 0.28 | 0.78 |
|  |  | residuals | 117 | 0.03 | 0.01 | 3.49 | 0.00 |
|  |  |  |  |  |  |  |  |
| *S. retusa* | H | snowmelt | 1 | 1522.97 | 1522.97 | 74.40 | 0.00 |
|  |  | site | 1 | 5.76 | 5.76 | 0.28 | 0.60 |
|  |  | residuals | 117 | 2394.88 | 20.47 |  |  |
|  | LA | snowmelt | 1 | 820.10 | 820.10 | 16.57 | 0.00 |
|  |  | site | 1 | 331 | 331 | 6.69 | 0.01 |
|  |  | residuals | 117 | 5792.20 | 49.51 |  |  |
|  | SLA | snowmelt | 1 | 142.33 | 142.33 | 13.65 | 0.00 |
|  |  | site | 1 | 192.96 | 192.96 | 18.50 | 0.00 |
|  |  | residuals | 117 | 1220.18 | 10.43 |  |  |
|  | log(LDMC) | snowmelt | 1 | 0.00 | 0.00 | 0.03 | 0.87 |
|  |  | site | 1 | 0.34 | 0.34 | 18.53 | 0.00 |
|  |  | residuals | 117 | 2.15 | 0.02 |  |  |
|  | LNC | snowmelt | 1 | 17.80 | 17.80 | 12.51 | 0.00 |
|  |  | site | 1 | 1.84 | 1.84 | 1.29 | 0.27 |
|  |  | residuals | 17 | 24.20 | 1.42 |  |  |
|  | LPC | snowmelt | 1 | 0.13 | 0.13 | 2.27 | 0.15 |
|  |  | site | 1 | 0.01 | 0.01 | 0.15 | 0.70 |
|  |  | residuals | 17 | 0.98 | 0.06 |  |  |
|  | N:P | snowmelt | 1 | 0.24 | 0.24 | 0.08 | 0.77 |
|  |  | site | 1 | 0.03 | 0.03 | 0.01 | 0.91 |
|  |  | residuals | 17 | 47.91 | 2.82 |  |  |
|  | SN | snowmelt | 1 | 4.61 | 4.61 | 3.48 | 0.06 |
|  |  | site | 1 | 0.87 | 0.87 | 0.65 | 0.42 |
|  |  | residuals | 105 | 138.89 | 1.32 |  |  |
|  | SM | snowmelt | 1 | 0.00 | 0.00 | 0.03 | 0.86 |
|  |  | site | 1 | 0.00 | 0.00 | 1.11 | 0.29 |
|  |  | residuals | 105 | 0.28 | 0.00 |  |  |
|  |  |  |  |  |  |  |  |
| *V. alpina* | log(H) | snowmelt | 1 | 0.34 | 0.34 | 2.89 | 0.09 |
|  |  | site | 2 | 18.91 | 9.46 | 79.95 | 0.00 |
|  |  | residuals | 176 | 20.58 | 0.12 |  |  |
|  | log(LA) | snowmelt | 1 | 0.19 | 0.19 | 1.61 | 0.21 |
|  |  | site | 2 | 4.98 | 2.49 | 20.67 | 0.00 |
|  |  | residuals | 176 | 20.97 | 0.12 |  |  |
|  | log(SLA) | snowmelt | 1 | 0.19 | 0.19 | 8.69 | 0.00 |
|  |  | site | 2 | 0.10 | 0.05 | 2.31 | 0.10 |
|  |  | residuals | 176 | 3.72 | 0.02 |  |  |
|  | LDMC | snowmelt | 1 | 13661 | 13661 | 30.45 | 0.00 |
|  |  | site | 2 | 914 | 457 | 1.02 | 0.36 |
|  |  | residuals | 176 | 78513 | 449 |  |  |
|  | LNC | snowmelt | 1 | 7.01 | 7.01 | 0.62 | 0.44 |
|  |  | site | 2 | 16.00 | 8.00 | 0.71 | 0.50 |
|  |  | residuals | 26 | 291.65 | 11.22 |  |  |
|  | LPC | snowmelt | 1 | 0.10 | 0.10 | 0.72 | 0.40 |
|  |  | site | 2 | 5.42 | 2.71 | 20.46 | 0.00 |
|  |  | residuals | 26 | 3.44 | 0.13 |  |  |
|  | N:P | snowmelt | 1 | 22.45 | 22.45 | 4.38 | 0.05 |
|  |  | site | 2 | 219.94 | 109.97 | 21.44 | 0.00 |
|  |  | residuals | 26 | 133.38 | 5.13 |  |  |
|  | SN | snowmelt | 1 | 1312 | 1312 | 6.00 | 0.02 |
|  |  | site | 2 | 1625 | 813 | 3.72 | 0.03 |
|  |  | residuals | 176 | 38481 | 219 |  |  |
|  | SM | snowmelt | 1 | 0.00 | 0.00 | 2.42 | 0.12 |
|  |  | site | 2 | 0.01 | 0.01 | 42.58 | 0.00 |
|  |  | residuals | 176 | 0.03 | 0.00 |  |  |
|  |  |  |  |  |  |  |  |
| *G. supinum* | H | snowmelt | 1 | 134.51 | 134.51 | 10.94 | 0.00 |
|  |  | site | 1 | 24.80 | 24.80 | 2.02 | 0.16 |
|  |  | residuals | 117 | 1438.95 | 12.30 |  |  |
|  | log(LA) | snowmelt | 1 | 0.52 | 0.52 | 4.38 | 0.04 |
|  |  | site | 1 | 0.18 | 0.18 | 1.50 | 0.22 |
|  |  | residuals | 117 | 13.84 | 0.12 |  |  |
|  | log(SLA) | snowmelt | 1 | 0.04 | 0.04 | 1.96 | 0.16 |
|  |  | site | 1 | 0.67 | 0.67 | 31.92 | 0.00 |
|  |  | residuals | 117 | 2.47 | 0.02 |  |  |
|  | LDMC | snowmelt | 1 | 8991 | 8991 | 10.88 | 0.00 |
|  |  | site | 1 | 55947 | 55947 | 67.71 | 0.00 |
|  |  | residuals | 117 | 96678 | 826 |  |  |
|  | LNC | snowmelt | 1 | 14.26 | 14.26 | 3.99 | 0.06 |
|  |  | site | 1 | 0.35 | 0.35 | 0.10 | 0.76 |
|  |  | residuals | 17 | 60.71 | 3.57 |  |  |
|  | LPC | snowmelt | 1 | 0.02 | 0.02 | 0.21 | 0.66 |
|  |  | site | 1 | 3.28 | 3.28 | 35.29 | 0.00 |
|  |  | residuals | 17 | 1.58 | 0.09 |  |  |
|  | log(N:P) | snowmelt | 1 | 0.11 | 0.11 | 2.59 | 0.13 |
|  |  | site | 1 | 0.69 | 0.69 | 16.40 | 0.00 |
|  |  | residuals | 17 | 0.72 | 0.04 |  |  |
|  | SN | snowmelt | 1 | 3910.20 | 3910.20 | 59.36 | 0.00 |
|  |  | site | 1 | 2660.20 | 2660.20 | 40.39 | 0.00 |
|  |  | residuals | 117 | 7706.90 | 65.90 |  |  |
|  | SM | snowmelt | 1 | 0.00 | 0.00 | 19.65 | 0.00 |
|  |  | site | 1 | 0.01 | 0.01 | 68.47 | 0.00 |
|  |  | residuals | 117 | 0.02 | 0.00 |  |  |
|  |  |  |  |  |  |  |  |
| *L. alpina* | H | snowmelt | 1 | 130 | 130 | 3.57 | 0.06 |
|  |  | site | 1 | 153 | 153 | 4.20 | 0.04 |
|  |  | residuals | 117 | 4258.70 | 36.40 |  |  |
|  | LA | snowmelt | 1 | 2112 | 2112 | 5.37 | 0.02 |
|  |  | site | 1 | 251 | 251 | 0.64 | 0.43 |
|  |  | residuals | 117 | 46043.00 | 393.53 |  |  |
|  | log(SLA) | snowmelt | 1 | 1.63 | 1.63 | 33.58 | 0.00 |
|  |  | site | 1 | 0.47 | 0.47 | 9.73 | 0.00 |
|  |  | residuals | 117 | 5.68 | 0.05 |  |  |
|  | LDMC | snowmelt | 1 | 28981 | 28981 | 53.54 | 0.00 |
|  |  | site | 1 | 961 | 961 | 1.77 | 0.19 |
|  |  | residuals | 117 | 63328 | 541 |  |  |
|  | LNC | snowmelt | 1 | 2.05 | 2.05 | 1.34 | 0.26 |
|  |  | site | 1 | 0.54 | 0.54 | 0.36 | 0.56 |
|  |  | residuals | 17 | 26.02 | 1.53 |  |  |
|  | log(LPC) | snowmelt | 1 | 0.30 | 0.30 | 11.20 | 0.00 |
|  |  | site | 1 | 0.17 | 0.17 | 6.37 | 0.02 |
|  |  | residuals | 17 | 0.45 | 0.03 |  |  |
|  | N:P | snowmelt | 1 | 11.58 | 11.58 | 5.73 | 0.03 |
|  |  | site | 1 | 5.49 | 5.49 | 2.72 | 0.12 |
|  |  | residuals | 17 | 34.35 | 2.02 |  |  |
|  | SN | snowmelt | 1 | 9442 | 9442 | 29.78 | 0.00 |
|  |  | site | 1 | 5602 | 5602 | 17.67 | 0.00 |
|  |  | residuals | 110 | 34875 | 317 |  |  |
|  | SM | snowmelt | 1 | 0.02 | 0.02 | 4.19 | 0.04 |
|  |  | site | 1 | 0.00 | 0.00 | 0.11 | 0.75 |
|  |  | residuals | 110 | 0.49 | 0.00 |  |  |
|  |  |  |  |  |  |  |  |
| *P. viviparum* | log(H) | snowmelt | 1 | 0.06 | 0.06 | 1.41 | 0.24 |
|  |  | site | 1 | 2.98 | 2.98 | 72.67 | 0.00 |
|  |  | residuals | 117 | 4.80 | 0.04 |  |  |
|  | LA | snowmelt | 1 | 11676 | 11676 | 7.04 | 0.01 |
|  |  | site | 1 | 15416 | 15416 | 9.30 | 0.00 |
|  |  | residuals | 117 | 193925 | 1657.50 |  |  |
|  | SLA | snowmelt | 1 | 21.61 | 21.61 | 6.95 | 0.01 |
|  |  | site | 1 | 0.45 | 0.45 | 0.15 | 0.70 |
|  |  | residuals | 117 | 363.59 | 363.59 |  |  |
|  | LDMC | snowmelt | 1 | 19291 | 19291 | 29.49 | 0.00 |
|  |  | site | 1 | 4402 | 4402 | 6.73 | 0.01 |
|  |  | residuals | 117 | 76548 | 654.30 |  |  |
|  | LNC | snowmelt | 1 | 25.02 | 25.02 | 3.85 | 0.07 |
|  |  | site | 1 | 0.70 | 0.70 | 0.11 | 0.75 |
|  |  | residuals | 17 | 110.43 | 6.50 |  |  |
|  | LPC | snowmelt | 1 | 2.93 | 2.93 | 14.24 | 0.00 |
|  |  | site | 1 | 1.89 | 1.89 | 9.15 | 0.01 |
|  |  | residuals | 17 | 3.50 | 0.21 |  |  |
|  | N:P | snowmelt | 1 | 35.58 | 35.58 | 10.75 | 0.00 |
|  |  | site | 1 | 35.25 | 35.25 | 10.66 | 0.00 |
|  |  | residuals | 17 | 56.24 | 3.31 |  |  |

Figure S1. Maps of the detailed locations of the experimental snowbed sites (a = Forcella Travenanzes; b = Pale di San Martino; c = Val Martello, d = Passo Gavia). The black circles indicate the precise location of each site, each of them comprising early and late snowmelt areas. The increasing darkness of the gray color corresponds to the increasing steepness of the slope (<http://cran.r-project.org/package=leafletR>, Graul 2016).


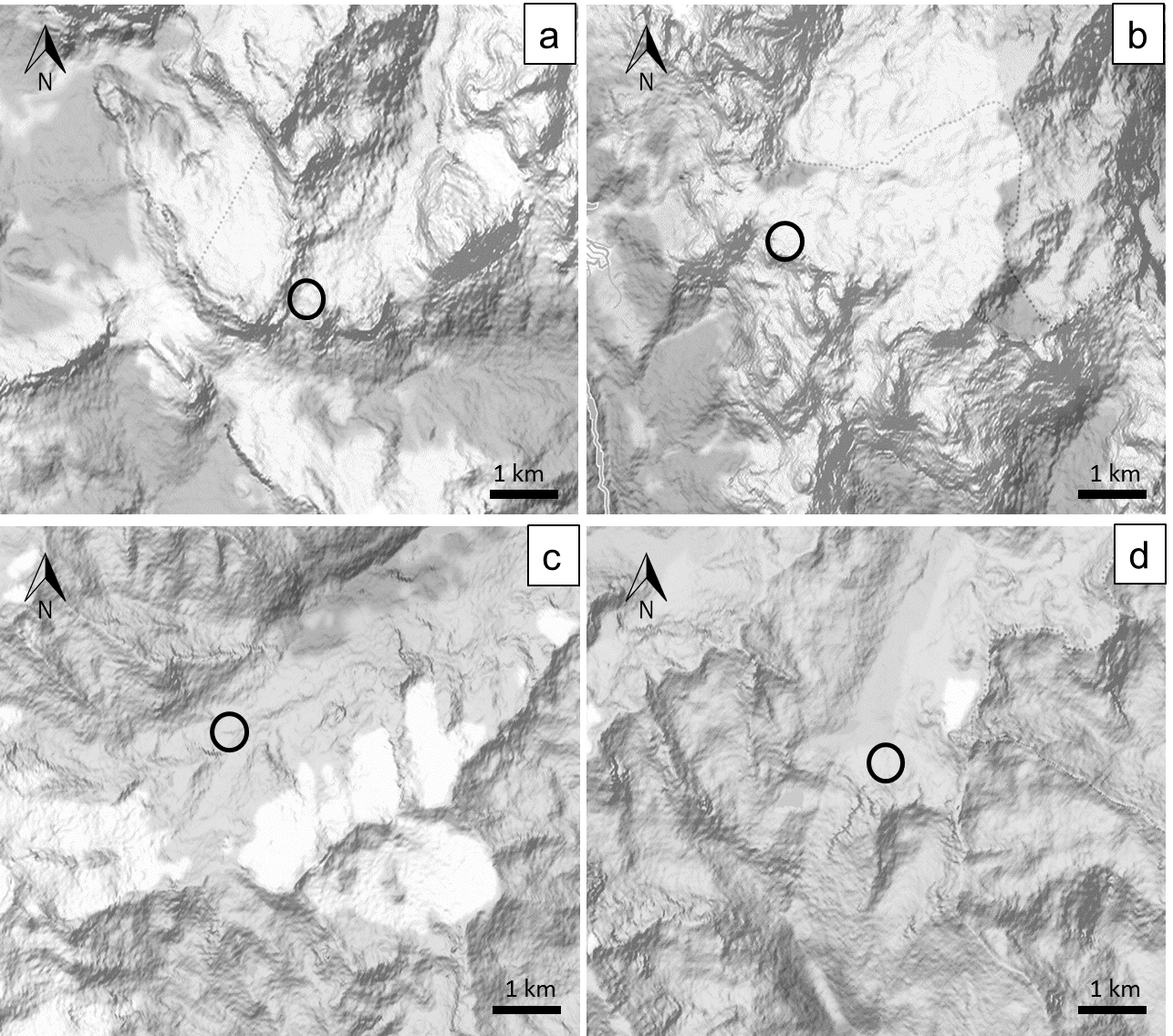


Figure S2. Mean temperatures and mean precipitation of winter months (November, December, January, February, March) from winter 2005/2006 to winter 2015/2016 (in red) from climatic stations close to the study sites (a, b= temperature and precipitation measured at Passo Falzarego for Forcella Travenanzes; c, d = temperature and precipitation measured at Passo Rolle for Pale di San Martino; e, f = temperature and precipitation measured at Solda-Madriccio for Val Martello; g, h = temperature and precipitation measured at Passo Tonale for Passo Gavia). Data for precipitation in Val Martello were available only from winter 2009/2010. Data obtained from Passo Tonale and Passo Rolle belong to the Provincia Autonoma di Trento, Dipartimento Protezione Civile e Infrastrutture, Servizio Prevenzione Rischi, Ufficio Previsioni e Pianificazione. Data from Solda-Madriccio station belong to the Province of Bolzano. Data from Passo Falzarego station belong to the Agenzia Regionale per la Prevenzione e Protezione Ambientale del Veneto.


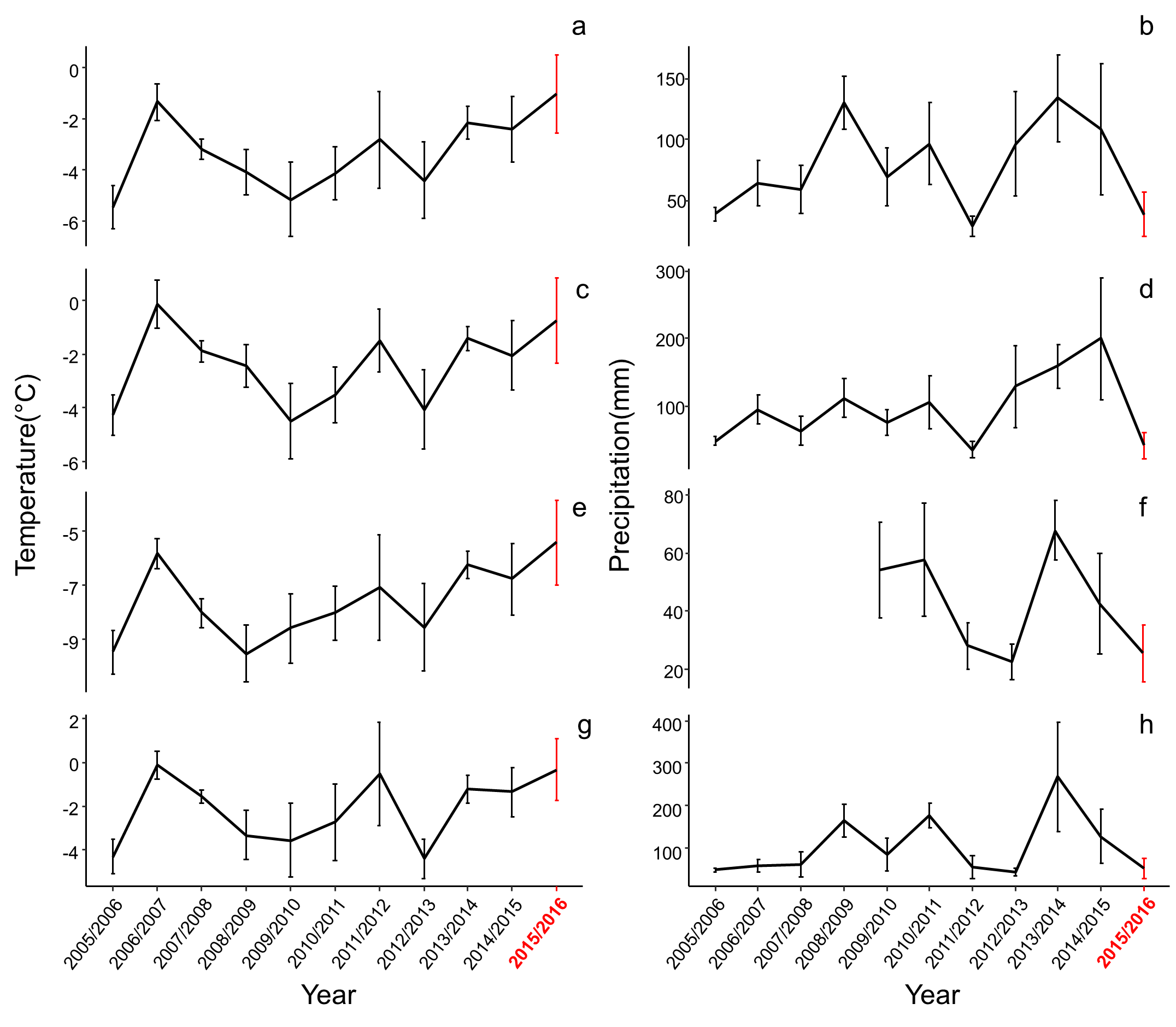


References

Díaz, S., Kattge, J., Cornelissen, J. H. C., Wright, I. J., Lavorel, S., Dray, S., et al. (2016). The global spectrum of plant form and function. *Nature* 529, 167–171. doi:10.1038/nature16489.

Graul, C. (2016). leafletR: Interactive Web-Maps Based on the Leaflet JavaScricpt Library. R package version 0.4-0. Available at: <http://cran.r-project.org/package=leafletR>.

Güsewell, S. (2004). N : P ratios in terrestrial plants : variation and functional significance. *New Phytol.* 164, 243–266. doi:[10.1111/j.1469-8137.2004.01192.x](https://doi.org/10.1111/j.1469-8137.2004.01192.x).

Güsewell, S., and Koerselman, W. (2002). Variation in nitrogen and phosphorus concentrations of wetland plants. *Perspect. Plant Ecol. Evol. Syst.* 5, 36-61. doi:[10.1078/1433-8319-0000022](https://doi.org/10.1078/1433-8319-0000022).

Kleyer, M., Bekker, R. M., Knevel, I. C., Bakker, J. P., Sonnenschein, M., Poschlod, P. et al. (2008). The LEDA Traitbase: a database of life-history traits of the Northwest European flora. *J. Ecol.* 96, 1266-1274. doi: 10.1111/j.1365-2745.2008.01430.x.

Leishman, M. (2001). Does the seed size/number trade-off model determine plant community structure? An assessment of the model mechanisms and their generality. *Oikos*. 93, 294-302. doi: [10.1034/j.1600-0706.2001.930212.x](https://doi.org/10.1034/j.1600-0706.2001.930212.x).

Pérez-Harguindeguy, N., Díaz, S., Garnier, E., Lavorel, S., Poorter, H., Jaureguiberry, P., et al. (2013). New handbook for standardised measurement of plant functional traits worldwide. *Aust. J. Bot.* 61, 167–234. doi:10.1071/BT12225.

Venable, D. L., and Brown, J. S. (1988). The selective interactions of dispersal, dormancy, and seed size as adaptations for reducing risk in variable environments. *Amer. Nat.* 131, 360-384. doi: 10.1086/284795.

Wellstein, C., Chelli, S., Campetella, G., Bartha, S., Galiè, M., Spada, F., et al. (2013). Intraspecific phenotypic variability of plant functional traits in contrasting mountain grasslands habitats. *Biodivers. Conserv.* 22, 2353–2374. doi:10.1007/s10531-013-0484-6.

Westoby, M. (1998). A leaf-height-seed ( LHS ) plant ecology strategy scheme. *Plant Soil* 199, 213–227.
